# Hardware Without Software: Teachers’ Cultural Silence and Menstrual Hygiene Management in Rural Bangladeshi Schools

**DOI:** 10.1002/puh2.70193

**Published:** 2026-02-09

**Authors:** Abdul Basit

**Affiliations:** ^1^ Department of Public Administration Shahjalal University of Science and Technology Sylhet Bangladesh

**Keywords:** institutional readiness, menstrual hygiene management (MHM), rural haor schools, *Shorom–Lojja* culture, silent infrastructure, teachers’ perception

## Abstract

In low‐income settings like Bangladesh, most menstrual hygiene management (MHM) initiatives are still limited to hardware, such as toilets, water, or waste disposal systems, but software aspects such as culture, attitudes, and institutional behaviors are neglected. This qualitative study aimed to analyze how teachers’ perspectives, social constraints, and institutional preparedness influence MHM implementation in schools in flood‐prone rural haor regions. Data were collected through in‐depth interviews and nonparticipant observations with 52 teachers (22 female, 30 male) in 11 secondary schools. Thematic analysis shows that, although menstruation is biologically recognized, it remains a socially taboo subject in the school environment. Male teachers, in particular, avoid discussing MHM in class due to the *Shorom–Lojja* (a culturally embedded shame–modesty norm) culture and fear of social stigma. This cultural software reduces the effectiveness of existing hardware in schools, resulting in the creation of a silent infrastructure. Institutional silence, lack of teacher training, and inadequate infrastructure exacerbate this deprivation. The research shows that achieving true menstrual equity requires a two‐pronged transformation: on the one hand, improving school hardware, and on the other, changing cultural and institutional software. Providing teachers with social recognition, training, and policy support can make the infrastructure effective. It is important to recognize MHM not only as an individual issue but also as an issue of dignity, education, and public health.

## Graphical abstract

This study reveals how cultural taboos, teacher discomfort, and inadequate school infrastructure collectively silence menstrual hygiene management education in rural haor schools of Bangladesh, contributing to student absenteeism and unmet health needs despite teachers’ awareness of its importance.

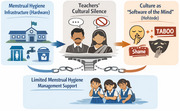

## 1 Background of the Study

Menstrual hygiene and sanitation are now global public health issues, being considered an important issue of education and human rights [1, 2]. Approximately 1.8 billion people go through menstruation every month [3], but many of them lack adequate knowledge, resources, and facilities to manage their periods in a dignified manner [4, 5]. Due to shame, prejudice, and poverty, girls cannot manage their periods safely [6], which results in them falling behind in school, work, and social life [7, 8]. Due to the lack of a supportive environment in schools, many girls are absent during their periods, which affects the quality of their education and mental health [9]. These problems are hindering the achievement of the Sustainable Development Goals (SDGs) on health, education, and gender equality [10, 11].

The World Health Organization (WHO) has called for menstruation to be considered a health and human rights issue [12]. But most efforts worldwide are still hardware dependent, such as providing toilets, water, soap, or disposal bins. However, the real challenge is often in people's knowledge, mindset, culture, and behavior [1, 13], which can be considered software in part. For example, only 39% of schools still teach menstrual hygiene, and less than one‐third of girls’ toilets have sanitary disposal bins [13]. In other words, even if the physical structure is in place, it is not effective if there is no social and psychological readiness.

Teachers can play a key role in meeting this hardware–software gap. They can help prepare students emotionally, approach menstruation naturally, and break down stigma [14]. However, in reality, most education systems in developing countries do not yet provide teacher training on menstrual hygiene management (MHM) or include it in their curricula [15, 16]. As a result, MHM is not discussed in classes, and many girls do not know anything about the topic before they enter puberty [7, 17]. Studies have shown that if teachers are trained, both school attendance and confidence in girls increase [18, 19].

In the context of Bangladesh, the hardware–software disparity is obvious. According to the 2018 Bangladesh National Hygiene Survey, only 36% of girls learned about the issue before their first menstruation. Only 39% of schools had water and soap in girls’ toilets, and 22% had sanitary bins [20]. Almost one‐third of girls miss school during their periods [21, 22]. The government has adopted several policies, such as the Eighth Five‐Year Plan (2020–2025) and the National MHM Strategy (2021), that address toilets, water, and menstrual education for girls in schools [23, 24]. However, these initiatives are largely limited in hardware development; software aspects, such as the attitudes of teachers and society, remain unprepared.

This reality is most acute in the rural haor region. Haors are low‐lying areas in northeastern Bangladesh that are flooded for about half of the year during the rainy season, including the districts of Sunamganj, Sylhet, Moulvibazar, Habiganj, Kishoreganj, Netrokona, and Brahmanbaria. Haor covers 1.99 million hectares and supports nearly 19.37 million people through fishing, rice cultivation, and other activities [25]. These areas are flooded every year, making it difficult to attend school, access water, and use toilets. In addition, society is relatively conservative, with talking openly about menstruation still considered “shameful” [26]. To date, most research on MHM in Bangladesh has focused on women's experiences, but research on teachers’ perspectives of menstruation is very limited. WHO [13] and Alam et al. [23] also report that only 1% of girls cite teachers as a source of information about menstruation. As a result, we still do not know how teachers themselves view the issue, how they respond, or how prepared schools are in terms of facilities, materials, and training. Thus, this study focuses on uncovering the neglected software dimensions of MHM, teachers’ perceptions, ethical dilemmas, and institutional preparedness in rural hair region of Bangladesh. Therefore, this study is not just a local social issue, but part of a larger public health and gender equity issue. This study connects the rural reality of Bangladesh with global MHM discussions. This study shows how cultural silence and inadequate institutional preparation together hinder girls’ participation in education. Therefore, the findings of this study are relevant not only for Bangladesh but also for all countries where education systems and societies still face the challenge of hardware–software integration of MHM.

## 2 Methods and Materials

This study adopted a qualitative approach to gain an in‐depth understanding of secondary school teachers’ perspectives, attitudes, and institutional preparedness for MHM in rural areas. This approach was chosen because it provides insights into participants’ lived experiences, social beliefs, and cultural contexts, which are often not captured by quantitative analyses. The study was conducted in two haor districts of Bangladesh, *Sunamganj* and *Netrokona*, which are regularly affected by natural disasters, such as floods and waterlogging, and are known to be among the most infrastructurally backward regions. A total of 10 secondary schools in these two districts were purposively selected. A total of 52 teachers from the selected schools participated, including 22 female and 30 male teachers. Teachers were selected purposively rather than including all teachers from each school. The selection criteria included a minimum of 3 years of teaching experience to ensure adequate institutional exposure, availability during the data collection period, and willingness to participate. Special emphasis was placed on including female teachers whenever possible because of their direct relevance to MHM. However, as most schools do not have a single female teacher, the number is relatively small. The sample size was determined on the basis of the principle of data saturation rather than numerical representativeness. Interviews were conducted until no new themes emerged. As a qualitative study, emphasis was placed on depth, contextual understanding, and interpretive richness rather than breadth. Data were collected through in‐depth interviews using a semi‐structured checklist that was iteratively refined during fieldwork. Credibility was established through piloting, triangulation with observational data, and iterative revision. Instead of conducting a formal back‐translation of the checklist, the researcher ensured semantic equivalence through fluency in the local language, ongoing adjustments, and verbatim transcription prior to translation. Each interview lasted for an average of 40–45 min. Most interviews were conducted by the researcher, who was fluent in the regional language of the local haor area and allowed trust and rapport with the participants. However, in some cases, female teachers may feel uncomfortable talking openly with a male researcher; therefore, interviews were conducted by a trained female data collector so that participants felt comfortable and could honestly share their experiences. The researcher used nonparticipant observations to assess the schools’ institutional readiness. Through this, the researcher observed the condition of the school's toilets, access to water and soap, separate areas for girls, waste disposal systems, and the overall hygiene environment. The observational data collected in this way were analyzed along with the interview data to obtain a comprehensive idea of the actual preparedness of the school.

The interviews were audio‐recorded with the permission of the participants and later transcribed verbatim into Bengali. They were then translated into English, and thematic analysis was performed. Key themes were extracted through open coding, theme identification, and recurrent pattern analysis of the data. Verbatim quotations in the findings were used to illustrate recurrent themes rather than isolated views. Quotations were selected on the basis of their analytical relevance and consistency across multiple interviews. To ensure the reliability and validity of the study, the primary results were shared with at least one teacher from each of the six schools to ensure that the researchers’ interpretations were consistent with their opinions and that no misinterpretations were made. In addition, triangulation was performed between the interview and observation data to increase the credibility of the results. Ethical standards were strictly adhered to during each stage of the study. The participants were informed in advance of the purpose of the study, confidentiality, and the right to withdraw at any time, and their written consent was obtained. The interviews were conducted in a separate quiet room at the school so that the participants felt comfortable. All information was kept confidential, and pseudonyms were used in the report. Gender sensitivity, cultural respect, and emotional comfort of the participants were maintained throughout the process.

## 3 Findings

### 3.1 Awareness and Understanding of MHM

The study found that both male and female teachers had a limited and fragmented understanding of menstruation as a biological process. Many male teachers view menstruation as a subject outside their purview. This is shaped by long‐standing gender norms that place the subject of menstruation exclusively feminine. One male teacher (Age‐38) reflected,

I don't know much about it (menstruation), never tried to find out myself. All I know is that this happens to women every month. We have never learned about it properly. Even in teacher training, no one mentioned such issues. It seems to be a women's problem, not something for us men to discuss.

This distance indicates a knowledge gap as well as socially internalized isolation from menstrual health education. Female teachers also reported that their first understanding of menstruation did not come through scientific explanations or from any recognized source, but through informal, often restrictive social and family instructions. As one female teacher (Age‐30) recalled,

I learned about it from my elder sister as a child. She just told me to keep quiet and not touch anything. I did not understand what was going on. Even now, I cannot clearly explain the biological aspect (of menstruation) to my students. That childhood education is so ingrained in me that I still treat it as a secret.

These early social messages of silence and secrecy continue to influence their current teaching practices, indicating that the culture of secrecy surrounding menstruation persists in professional spaces and continues to influence. A recurring expression across narratives is a deep sense of discomfort when confronted with menstrual discussions in an educational context. One male participant (Age‐41) admitted,

To be honest, I am uncomfortable even hearing anything about this. I have two daughters, but my wife handles these things. I keep quiet about these things. I feel ashamed to talk openly about such things.

Another female teacher (Age‐36) shared,

Even among us female teachers, we do not talk about it. Once I tried discussing it with some girl students privately, but my colleagues asked why I was talking about such ‘dirty topics.’ After that, I stopped.

Teachers also had feelings of guilt and helplessness, especially when they saw students suffering during menstruation, but felt unable to help students due to a lack of training or fear of social judgment. One experienced male teacher (Age‐48) reflected,

I have been teaching for 20 years, but no one has ever asked us to know or teach about menstruation. Sometimes, I feel guilty. The girls suffer in silence, and I cannot help them, give them any guidance, because I have never been given any guidance.

These observations suggest that many teachers feel subtle but persistent discomfort when discussing menstruation in the classroom. This discomfort is not just a personal feeling; it reflects deep cultural norms and social taboos surrounding menstruation. Such professional discomfort may conflict with their roles as teachers, as it may limit open communication with students, reduce teaching effectiveness, and hinder the creation of a supportive learning environment for students.

When asked if they were confident in explaining the topic of menstruation to students, most respondents admitted that they deliberately avoided the topic. A male teacher (Age‐29) disclosed,

Every time I try to explain anything about menstruation, my throat goes dry. I'm afraid the students will laugh, or the parents will complain that I'm talking rudely.

Even when girls directly asked questions about menstrual health management, the teachers relied on avoidance as a defensive strategy. A male teacher (Age 40) said,

Sometimes girls ask questions in physical education class, but I just say, ‘You can ask the female teacher.’ It's safer for me. I don't want trouble.

Female teachers expressed a similar pattern of silence shaped by the fear of social interpretation. As one (Age‐34) mentioned,

I once thought of taking a separate session for girls, but I hesitated. What if boys hear from the next class? What if the other teachers heard? Rumors spread rapidly in villages. I did not dare.

Thus, menstruation remains intellectually recognized, yet socially unapproachable, within the school context, hindering the normalization of MHM education.

### 3.2 Attitudes and Beliefs Surrounding Menstruation

Although some teachers acknowledged menstruation as a natural biological process, their narratives revealed profound conflicts shaped by the cultural norms of shame and moral respect. One male participant (Age‐48) said,

Menstruation A normal process. However, from childhood, we are taught that women should not talk about personal matters. It seems impolite to even mention this word. So, even though I understand it is biological, my heart flutters when I hear about it. It's not easy to go against what society has built into us over generations.

This reflects how biological incomprehensibility exists in parallel with culturally enforced silence, resulting in ambivalent acceptance rather than active acceptance. Teachers describe that girls’ first menstruation in rural haor communities is often viewed as a matter of privacy rather than a stage of healthy development. As one female respondent (Age‐33) recalled,

When a girl in our village gets her first period, the family hides it. They do not let her go out or even go to school for a few days. It is seen as a moment of shame rather than a normal process of growing up. I remember my own mother, telling me not to talk to anyone about it. That memory still affects me’. It's hard for me to see it as normal, even though I know better now.

These practices demonstrate how menstrual stigma is reproduced in family structures before being reinforced in the school environment. Several teachers have explicitly described menstruation as a *nishiddho bishoy* (a forbidden topic) within public discourse. One male participant (Age‐44) stated,

In our area, you can talk openly about politics, religion, even adult films but not about this topic. Talking openly about menstruation risks losing respect. Whether we admit it or not, it remains a taboo subject, stronger than any rule on paper.

This fear is not abstract. This was further reinforced by actual incidents of social punishment. One of the most shocking narratives came from a female teacher who recounted a recent incident involving a male colleague. One male teacher (Age‐40) shared,

One of our male colleagues discussed menstrual health management in the class one day. The next day, the students (boys) of the school complained to the headmaster that “Sir is talking indecent in the class.” A few days later, a colleague was kicked out of the rented house where he lived. And the local people disciplined him saying that ‘if he talks like this in the class in the future, he will be beaten out of the area.’ In this situation, whether I am a female teacher or a male teacher, how can I talk about these things in the class?!

The idea of teachers, especially male teachers, discussing MHM in the classroom was met with fear of community backlash. Some female teachers expressed caution and expressed silence as a socially protective strategy. One female teacher (Age‐33) said,

Our society sees menstruation as shameful. If I try to teach this, people may question my character. They might say, “Why is she talking about these dirty things?”

It shows how teachers navigate between their educational roles and their social identities in a conservative rural context, where the concept of community acts as a powerful regulator. Internal conflict was evident among many teachers, especially female teachers, who recognized the pedagogical need for MHM awareness but felt constrained by societal expectations. One female participant (Age‐38) narrated,

Sometimes I feel very self‐conflicted. As a teacher, I know that ignorance should be broken through education. It is the teacher's main responsibility. However, as a woman living here, I know how difficult it is to go against the rules. Even when a girl faints during menstruation, we cannot explain the real reason. We simply say that she is sick. The silence is heavy, hard to accept, but it is safer than being accused of spreading obscenity.

This tension highlights the emotional burden on teachers, who are aware of their responsibilities but are constrained by the fear of moral judgment. On the other hand, different opinions were observed among some teachers. For many teachers, menstruation lay outside what they considered the legitimate scope of school education. This was evident in recurring statements that positioned menstrual knowledge as the responsibility of the family rather than educational institutions. As one male participant (Age‐47) clearly stated,

I find it uncomfortable to teach about menstruation in school. Girls should learn about it from their families, because it is a very personal matter.

Another male (Age‐31) echoed the same view, saying,

It is better for mothers to explain these things to their families’. It is not the place to teach them in school.

These perspectives reflect a strong cultural belief that menstruation belongs to the private domain of the household, particularly under the moral authority of mothers, rather than within the public discourse on schooling. Teachers also drew symbolic boundaries around what counts as appropriate educational content, often positioning menstruation outside moral and pedagogical legitimacy. One male participant (Age‐48) explained,

Even though menstruation is a physiological subject, there should be a limit. The job of the school is to teach morals, not to discuss the body.

This illustrates an underlying moral hierarchy where academic or religious instruction is considered respectable, whereas discussions involving the body, especially female bodily functions, are seen as transgressive within the school space.

### 3.3 Institutional Readiness and School Environment

Findings revealed that schools in the haor region lacked adequate infrastructure, administrative support, and institutional guidelines to address the menstrual hygiene needs of adolescent girls. Teachers consistently reported that the basic amenities required for menstrual management, such as clean toilets, access to water, and personal space, were absent or ineffective. One female teacher (Age‐33) described,

We have only one toilet for all girl students, and it is often dirty, most of the time there is no water. During monsoons, when haor floods, it becomes difficult to even reach the toilet as water surrounds the building. Girls avoid using it and are forced to stay at home when they menstruate.

The absence of private changing spaces led to emotional distress and absenteeism among girls. One female teacher (Age‐30) added,

If a girl has an accident during menstruation, she feels humiliated because there is no proper place to clean or change clothes. Other students may have made fun of her.

Another male teacher (Age‐34) added,

The school administration does not take it as a priority. The focus is always on results, tests, and attendance. Menstruation is never seen as something that needs planning or policies; it is seen as an inconvenience, not as a real health concern.1

In some cases, toilet security was insufficient. One female teacher (Age‐33) shared,

Our school building is old, and the toilets are often used by both boys and girls. The door does not even lock properly. Imagine the discomfort a teenage girl has to experience during her period! Many girls don't go to school for three or four days every month.

This indicates that infrastructural neglect is a direct cause of menstrual absenteeism, a concern that is institutionally unacknowledged. Teachers also reported that there were no formal policies, emergency supplies, or teacher‐training modules for MHM. One male participant (Age‐40) shared,

There are no sanitary pads in our school, or any guidance on what to do if a girl needs help. Everything is not governed by policy, but by informal empathy.

The lack of a structured system resulted in monthly management being limited to the individual and dependent on chance encounters with supportive female teachers rather than institutional responsibility. During the monsoon months, the situation worsens as the schools are partially surrounded by water, making it difficult for menstruating girls to move. One female teacher (Age‐39) noted,

During floods, the school becomes like an island. Girls avoid coming because they fear getting stained or uncomfortable and there is no place to change clothes.

Despite these repeated challenges, teachers reported no discussion or recognition from the school administration, indicating that menstrual hygiene remains invisible during formal planning and infrastructure development. To complement the interviews, nonparticipant observation was conducted to assess the school infrastructure. The following table summarizes the readiness indicators across the six schools: Table 1.

**TABLE 1:** Institutional readiness and school environment.

Observation indicators	Schools with facility (*n*)	Schools without facility (*n*)
Separate toilet for female students	8	2
Toilet easily accessible to girls (location, distance, safety)	2	8
Toilet ensures sufficient privacy (lockable doors, enclosed spaces)	2	8
Reliable water source inside or near toilet	1	9
Soap or handwash available after use	1	9
Private space for changing menstrual materials	0	10
Covered waste bin in girls’ toilets	0	10
Waste bin regularly cleaned or emptied	0	10
Safe and hygienic disposal system for sanitary pads	0	10
Toilet and surrounding area clean and odor‐free	0	10
Separate water container/provision during menstruation	0	10
Rest room/sick room for girls during menstruation	0	10
Arrangement for emergency sanitary pad supply	0	10
Designated person responsible for toilet maintenance	2	8

*Source:* Nonparticipant observation by the researcher.

The observation matrix confirmed that infrastructural readiness for MHM was critically low. Only one school ensured minimal privacy, and none provided water, soap, changing spaces, or disposal systems. The absence of emergency supplies or a responsible authority for maintenance indicates that menstrual hygiene is structurally unacknowledged within school governance. This institutional neglect reinforces the silence expressed by teachers, demonstrating how cultural and infrastructural barriers sustain menstrual exclusion in educational spaces (see Supporting Information Table S1 for a summary of themes and subthemes).

## 4 Discussion

The findings of this study reveal that teachers in rural Bangladesh, including the haor region, work within an educational environment where menstruation is biologically recognized but socially censored. Although menstruation is included in national educational frameworks as part of adolescent health, its classroom articulation is constrained by sociocultural norms. Similar to other studies in rural Bangladesh and South Asia [27, 28, 29, 30], and even developed countries [31], this study confirms that teachers internalize menstrual silence. *Shorom–Lojja* (*Shorom–Lojja* refers to a culturally enforced norm of modesty and shame that restricts open discussion of female bodily matters in rural Bangladesh) culture, a deeply embedded moral order that frames bodily matters, specifically female reproductive functions, as bound by shame and clearly socially risky. Under such ethical regulations, the teacher's role becomes a site of surveillance by community members, where stepping outside recognized academic subjects invites suspicion and respectable risk.

Contrary to the common discourse that primarily locates the stigma of menstruation among adolescent girls [32, 33], this study shows that teachers themselves are subject to similar cultural concerns. Evidence from other Bangladeshi studies [23, 34, 35] indicates that teachers, especially males, avoid engaging in menstrual management education for fear of being perceived as immodest or morally transgressive. In haor's context, this concern is exacerbated by tight‐knit community settings, where teachers are part of the same network that regulates student behavior. Community expectations provide mothers with menstruation education, making it a private and domestically controlled issue. This belief is consistent with findings from Nepal and Uganda, where menstrual knowledge is culturally positioned as “women's talk,” excluding male teachers from legitimate involvement [36, 37]. The study also found that avoiding teachers is not just a reflection of discomfort but a defensive strategy against potential social disapproval. Comparable fieldwork in northern Bangladesh [38, 39] documented cases in which teachers faced community backlash to address menstruation, reinforcing silence as a safe professional posture. In our study area, a documented case of a punitive community response to a teacher who attempted to address menstrual hygiene demonstrates that fear is not hypothetical but is constructively produced through informal processes of social discipline. This aligns with Sommer, Hirsch et al. [40] and Sommer, Ackatia‐Armah et al. [41], who argue that school‐based menstruation interventions fail not only because of resource gaps but also because of moral surveillance of teachers, which remains controversial in the policy framework.

Existing literature on MHM in Bangladesh often focuses on girls’ experiences. This study highlights the teacher as a constrained actor within the same cultural matrix, caught between educational responsibilities and community‐imposed moral boundaries. This extends the current discourse on menstrual health to a policy design that places responsibility on teachers without providing solutions. Social judgment and reputational vulnerability risk reinforcing rather than breaking the silence. Similar observations have been made in global analyses where teachers request institutional protection before engaging with stigmatized content [30, 42]. The absence of administrative approval or clear guidance in the schools studied confirms this gap. Institutional preparedness was critically lacking, with no structural arrangements for emergency pad provision, disposal systems, or changing personal spaces. This reflects the UNICEF Bangladesh (2018) national assessment, which reported that less than 20% of rural schools provide adequate monthly facilities [20]. In haor schools, infrastructural neglect intersects with environmental uncertainty. Studies on disaster‐prone areas in Bangladesh [26, 32] show that during floods, girls’ mobility is more limited, and school wash systems fail completely. Current findings confirm that during monsoon floods, girls are either absent from school or suffer humiliation due to a lack of personal sanitation, reinforcing absenteeism. It links directly to regional studies that show that flood‐exposed schools require period‐related adaptation, a dimension often overlooked in mainstream WASH policy.

The interaction between cultural taboos and infrastructural scarcity explains that MHM exclusion is not just a matter of awareness. Teachers did not feel empowered to raise menstrual issues because there was no structured mechanism to address them. As noted in the evaluation of WASH‐in‐school programs [13], discussions about menstruation without collateral support can lead to shame rather than empowerment. Haor's context exacerbates this dynamic: Without water, disposal units, or privacy, teachers’ engagement with MHM becomes symbolic and practically futile. The global MHM discourse is increasingly shifting toward a hygiene‐based framing rights‐based menstrual health framework [1, 40], emphasizing dignity and institutional accountability rather than individual coping. This study demonstrates support for the framework that teacher discourse, school infrastructure, and community ethics act as a combined regulatory system. In this sense, teachers are not merely uninformed but are constructively discouraged from engaging, reflecting Hennegan et al. [28]. This can also be best explained by Hofstede's concept of culture as “software of the mind” [43]. Existing literature has shown that MHM policies in rural Bangladesh have prioritized hardware interventions, toilets, pads, and drainage units, whereas cultural software that regulates teachers’ behavior has been neglected. In haor's schools, the *Shorom–Lojja* culture serves as an internal moral code, programming teachers to avoid menstruation‐related discussions to protect their social reputations. This cultural software overrides policy expectations, implying that infrastructure alone cannot bring about changes. As a result, schools create “silent infrastructure” facilities that exist physically but remain socially inactive.

### 4.1 Limitations of the Study

This study has several limitations. First, formal back translation of the interview checklist was not conducted. Although semantic equivalence was ensured through researcher fluency, iterative checklist revision, and verbatim transcription, the absence of formal back translation may affect linguistic precision. Second, the study was conducted in rural schools in haor regions of Bangladesh; therefore, the findings are context‐specific and cannot be generalized to urban settings or other educational contexts.

## 5 Conclusion

This study showed that improving MHM in rural and haor‐area schools in Bangladesh requires more than just infrastructure measures. The central actors in the school environment (teachers) operate within a cultural framework shaped by *Shorom–Lojja* norms, where discussing menstruation risks moral judgment and social sanctions. As a result, even when schools partially address physiological needs, institutional silence persists, creating a “silent infrastructure” that fails to translate into meaningful support for girls. Findings revealed that teachers’ limited biological understanding, internal discomfort, and fear of community surveillance significantly influenced how MHM is addressed within schools. Moreover, haor‐specific challenges such as seasonal flooding limit access, making menstrual management more difficult for students. Without policy‐supported cultural permission, training, and protection for teachers, menstruation will continue to be treated as a personal matter rather than an educational concern. Therefore, achieving menstrual equity in schools requires dual transformation. Upgrading physical facilities and simultaneously reprogramming cultural software that governs educational practices. Only when teachers’ discourse is institutionally legitimized and socially protected can infrastructure be activated into real support. In this context, menstrual health needs to be recognized not just as a sanitation issue but as a matter of dignity, teaching, and institutional responsibility.

## Author Contributions

**Abdul Basit:** Conceptualization, Methodology, Investigation, Data Curation, Formal Analysis, Validation, Visualization, Writing – Original Draft Preparation, Writing – Review & Editing, Project Administration.

## Funding

The authors have nothing to report.

## Ethics Statement

The study has received appropriate ethical approval, which was granted by the SUST Research Ethics Board (SREB), Ref: SSS/PAD/004/010.

## Conflicts of Interest

The author declares no conflicts of interest.

## Supporting information




**Table S1**: Summary of Major Themes and Sub‐themes on Teachers’ Cultural Silence and Institutional Preparedness for Menstrual Hygiene Management in Rural Schools.

## Data Availability

The data that support the findings of this study are available on request from the corresponding author. The data are not publicly available due to privacy or ethical restrictions.
