# Supplementary material for: Hardware Without Software: Teachers’ Cultural Silence and Menstrual Hygiene Management in Rural Bangladeshi Schools
Source: Public Health Chall. 2026 Feb 9;5(1):e70193. doi: 10.1002/puh2.70193 (PMC12887436; doi:10.1002/puh2.70193)
Supplement: Supplementary file 1 — Table S1: Summary of Major Themes and Sub‐themes on Teachers’ Cultural Silence and Institutional Preparedness for Menstrual Hygiene Management in Rural Schools. [file PUH2-5-e70193-s001.docx]

**Supplementary Materials 1**

**Table:** Summary of Major Themes and Sub-themes on Teachers’ Cultural Silence and Institutional Preparedness for Menstrual Hygiene Management in Rural Schools

| **SL** | **Major Themes** | **Sub-themes** |
| --- | --- | --- |
| 1 | Limited Awareness and Knowledge of Menstrual Hygiene Management | Fragmented understanding of menstruation as a biological process; Menstruation viewed as a “women’s issue” by male teachers; Lack of formal training on MHM during teacher education; Reliance on informal and restrictive social learning |
| 2 | Cultural Silence and Professional Discomfort | Discomfort and embarrassment in discussing menstruation; Avoidance of MHM topics in classrooms; Fear of student reactions and parental complaints; Silence as a coping and protective strategy |
| 3 | Attitudes and Beliefs Shaped by Social Taboos | Menstruation perceived as shameful or morally inappropriate; Menstruation considered a private family matter rather than a school responsibility; Internal conflict between professional duty and social norms; Fear of loss of social respect and moral judgment |
| 4 | Gendered Constraints and Community Surveillance | Heightened risk for male teachers discussing MHM; Female teachers’ fear of character judgment; Community backlash and social punishment; Informal regulation of teachers’ behavior by local norms |
| 5 | Institutional Unpreparedness for Menstrual Hygiene Management | Inadequate sanitation facilities; Lack of privacy, water, and soap; Absence of disposal systems and emergency supplies; No formal policies or administrative guidelines on MHM |
| 6 | Impact of Infrastructure and Ecology on Menstrual Management | Poor toilet accessibility during floods; Increased absenteeism during menstruation; Emotional distress and humiliation among girls; Seasonal worsening of MHM challenges in haor contexts |
